# Varroa-Virus Interaction in Collapsing Honey Bee Colonies

**DOI:** 10.1371/journal.pone.0057540

**Published:** 2013-03-19

**Authors:** Roy M. Francis, Steen L. Nielsen, Per Kryger

**Affiliations:** Department of Agroecology, Science and Technology, Aarhus University, Slagelse, Denmark; Sheffield University, United States of America

## Abstract

Varroa mites and viruses are the currently the high-profile suspects in collapsing bee colonies. Therefore, seasonal variation in varroa load and viruses (Acute-Kashmir-Israeli complex (AKI) and Deformed Wing Virus (DWV)) were monitored in a year-long study. We investigated the viral titres in honey bees and varroa mites from 23 colonies (15 apiaries) under three treatment conditions: Organic acids (11 colonies), pyrethroid (9 colonies) and untreated (3 colonies). Approximately 200 bees were sampled every month from April 2011 to October 2011, and April 2012. The 200 bees were split to 10 subsamples of 20 bees and analysed separately, which allows us to determine the prevalence of virus-infected bees. The treatment efficacy was often low for both treatments. In colonies where varroa treatment reduced the mite load, colonies overwintered successfully, allowing the mites and viruses to be carried over with the bees into the next season. In general, AKI and DWV titres did not show any notable response to the treatment and steadily increased over the season from April to October. In the untreated control group, titres increased most dramatically. Viral copies were correlated to number of varroa mites. Most colonies that collapsed over the winter had significantly higher AKI and DWV titres in October compared to survivors. Only treated colonies survived the winter. We discuss our results in relation to the varroa-virus model developed by Stephen Martin.

## Introduction

Honey bees are important insects economically and ecologically. Large scale losses of managed honey bees in the recent years have perplexed beekeepers and bee researchers. Honey bee colonies in temperate climates that do not survive the winter are referred to as winter losses. These losses may be a result of natural causes or varroa infestation and viruses [1], [2], [3]. DWV in combination with varroa mites have been shown to reduce lifespan of winter bees [4]. Colony Collapse Disorder (CCD) is a syndrome describing the large-scale loss of managed honey bees worldwide first reported in 2006–2007 [5]. Rise in colony losses were quickly reported from several locations worldwide [6]. Several studies have investigated and reported various causes for this sudden decline is bees such as viruses [7], [8], varroa mites [9], microsporidean *Nosema spp.* [10], [11], [12], and pesticides [13], [14], [15]. Due to the lack of consensus on a possible causal agent of colony losses, it is being investigated extensively [16] and it is becoming clear that a single causal agent is difficult to identify and that causes are possibly multiple and complex [8], [17], [18], [19], [20]. A combination of varroa and viruses are now frequently implicated in collapsing colonies [9], [21], [22], [23].

The single greatest threat to honey bee populations worldwide is the recently introduced invasive mite *Varroa destructor* Andersen & Trueman. The life cycle of the varroa mites is tightly adapted to the development of the honey bees. Varroa mites are serious and devastating ectoparasites of the honey bee. During the phoretic phase, the varroa mites live on the bodies of honey bees and feed on their haemolymph. The symptoms arising out of heavy mite infestation is referred to as varoosis [24], [25]. The reproductive phase of varroa mites happens exclusively in the capped cells of developing bee pupae [26]. Several studies have documented the ill effects of varroa infestation on honey bees including reduced lifespan [27], decreased survivorship [28] and weight loss in drones [29].

Varroa mites are also efficient vectors for transmission of viral diseases [30]. The rise of viral infection in bees after the introduction of varroa mites into Europe has been reported quite extensively [31]. Several honey bee viruses have been reported to be transmitted by varroa mites including DWV [32], KBV [33], [34], SBV [35], ABPV [30] and IAPV [36]. Several extensive survey studies have been carried out to monitor virus and mite levels over an entire season [37], [38], [39]. Thus we know that mite population increases from spring to autumn during peak brood production in the colony [40]. Drone brood in particular accelerates the growth of the mite population. Hence, mite levels and subsequently viral prevalence are highest towards autumn. During winter, the small cluster of bees ceases brood production which restricts mite reproduction. However, the phoretic mites may shift host thereby transmitting viruses within the colony.

Given the complex interactions between honey bees, varroa mites and viruses, modelling approaches have been applied to understand dynamics in the hive [41], [42], [43]. The model proposed by Stephen Martin [44], predicts that the less virulent DWV would be highly prevalent in varroa-infested colonies whereas the more virulent ABPV should disappear as it rapidly kills its host. Putting this model to test requires improved sampling strategies for estimating varroa and virus prevalence. A few methods have been put forward to estimate varroa load at colony and apiary level [40], [45], [46] while no guidelines have been established for optimal sampling for virus prevalence. So far, most studies have tried to determine the viral titre in a sample of workers from colonies, however, this cannot be used to estimate the prevalence of virus-infected individuals in the colony. In particular, there exists a huge variation in viral titres amongst bees, ranging from uninfected individuals to carriers of billions of viral particles, rendering the interpretation of data from pooled samples cumbersome. A recent study of 293 colonies from 35 apiaries across three years in Hawaii [23], reported a combination of DWV and varroa as the destructive force behind collapsing colonies. DWV infection was studied in varroa-free colonies through the phase of varroa infestation. The DWV titre in varroa infested colonies were 10^10^ copies per bee compared to 10^4^ copies in varroa-free colonies. As the DWV titres increased, the genetic diversity of DWV decreased ultimately leading to a single high-virulent species.

Beekeepers use various acaricides to keep mite levels low and to avoid outbreak of viral diseases. Acaricides control mites through different pathways and their side-effect on the colony may vary [47], [48]. In Denmark, most popular chemical methods include organic acids (formic acid, lactic acid, oxalic acid) and pyrethroid flumethrin. Now that, there is considerable evidence to show that a combination of viruses along with varroa is playing an important role in colony health, monitoring the changing viral load in relation to mite number over a whole season is vital to understand the nature of this interaction. Viral analysis was carried out on subsamples to estimate the prevalence of virus infected bees in the colonies. This study investigated viral load in bees and varroa mites across 23 colonies from 16 apiaries under three treatment conditions over a year.

## Materials and Methods

### Sample collection and processing

Honey bee worker samples were procured from our own experimental hives and from the following Danish beekeepers: Aksel Jørgensen, Arne Jensen, Bent Larsen, Christian Petersen, Ditlev Bluhme, Flemming Thorsen, Gunner Borg, Jørgen Jørgensen, Karen Poulsen, Leif Johanssen, Orla Overby and Willy Svendsen. Samples were collected from 23 colonies in 15 apiaries (Fig. S5). The colonies were categorised based on the method of treatment used to control mite population. Category A included 11 colonies (A01 to A11) from seven apiaries which were treated using organic acids mostly formic acid and always oxalic acid. Formic acid is used in early autumn followed by oxalic acid in early winter. Category B included nine colonies (B01 to B09) from seven apiaries which were treated using the pyrethroid Flumethrin. The beekeepers use non-standardised treatment methods which result in diverse methods of application and treatment times. Category C included three experimental colonies (C01, C02 and C03) from one apairy which were not treated in any manner. These colonies were also not treated the year before. One colony C02 was removed in September, due to American foulbrood and hence, not sampled in October 2011. Seven colonies (A04, A05, A09, B09, C01, C02, C03) died over winter and were not sampled in April 2012.

Approximately 200 bees were collected from every colony in the first week of every month from April 2011 to October 2011 and again in April 2012. All bee samples were received alive after being transported by post in 400 ml plastic containers with breathing holes and sugar candy food in 1.5 ml vials. Live bees were used to ensure high quality RNA. The bees were anaesthetised with carbon dioxide gas for 15 min and shaken in a polythene bag to release mites. The bee numbers and mite counts were recorded and approximately 200 bees were split into 10 separate subsamples of maximum 20 bees each. The mites from each colony were pooled together as a separate sub-sample. If samples received were less than 200 bees, then subsamples were split to 10 bees or 15 bees. Samples with more than 200 bees were stored as extras. From these extra bees, six individuals from eight samples (four colonies from September and four colonies from October) were analysed individually to estimate prevalence of diseased bees. Samples were freeze-dried on a Heto LyoPro 6000 apparatus for 72 hours at pressure 0.05 hPa and temperature −80°C. After lyophilisation, all samples were stored at −80°C in 50 ml plastic bottles with tight fitting screw-caps.

### RNA extraction and cDNA conversion

Metal beads were added to the sample bottles and the samples were homogenized on a genogrinder 2000 for 1 min at 1500 rpm. A pinch (approximately 10–15 mg) of the crushed material was used to extract RNA. In a pool of 20 bees, a pinch would be approximately 0.40 bee. Total RNA was extracted using NucleoMag® 96 RNA Kit (Macherey-Nagel) on a Kingfisher Magnetic Extractor using a custom program following the manufacturer's guidelines. The extracted RNA (100 µl) was stored in 96 well plates at −80°C for further use. The RNA was transcribed to cDNA using High Capacity cDNA Reverse-Transcription Kit (Applied Biosystems (AB)). 10 µl of RNA was added to 10 µl cDNA master mix yielding a 20 µl cDNA solution. The incubation conditions were as recommended by the manufacturer: 10 min at 25°C, 120 min at 37°C and 5 min at 85°C. The cDNA solution was then diluted 10-fold in water and stored at −80°C.

### Real-time PCR

The three assays tested in this study are AKI, DWV and beta-actin. The ABPV-KBV-IAPV viruses were detected in a single assay using a single pair of primers referred to as ‘AKI’ primers [49]. Two sets of primers were used for each virus (AKI and DWV), referred to as outer primers and inner primers [39]. Primers used in this study are listed in Table S1. Standard PCR was carried out on previously known positive samples using outer primers to generate stock of positive controls. Ten ten-fold dilutions, prepared from these stocks and were used on every plate to estimate plate to plate variation. All samples and controls were tested using inner primers. RNase-free water was used as template for negative controls (NTC). The real-time polymerase chain reaction (qPCR) assays were carried out on an ABI PRISM 7600HT (AB) using SYBR Green DNA binding dye (AB). The volume for qPCR reactions was 12 µl with a final primer concentration of 0.4 µM. All reactions were loaded on optical 384 well PCR plates (ABgene) and run in replicates of two.

### Data processing and analysis

Eight dilutions for AKI, beta-actin4 and DWV3 were selected for standard curves and subsequent regression analysis based on their linearity within the dynamic range. The baseline was automatically set and a manual threshold of 0.19 was used for all control runs and test runs. Dissociation profiles for all reactions were visually examined and flagged. Data from the qPCR runs were analysed in R 2.15.1 [50] and Microsoft Excel™. Replicates showing coefficient of variation (CV) greater than 10% were flagged and replicates were examined and manually corrected if required. Samples which did not cross the threshold before cycle 40 were given a C_t_ value of zero or no virus. Samples with incorrect melting curve profile were given a C_t_ value of zero.

The slope and intercepts calculated from the standard curves were used to estimate DNA copies from known C_t_ values (Fig. S1). DNA concentration was converted to copies using equation [copies = (c×N)/M] where where c = concentration in g, N = avogrado's constant and M = molecular mass of the amplicon in Daltons. While converting to copies, copies near 1 were rounded to 0 or 1 as it is assumed there has to be one copy of the virus/beta-actin or none. Based on the standard curves, a C_t_ Value of 34 was chosen as a cut-off because the standard curves were no longer linear after cycle 34. Based on the regression, cycle 34 corresponds to 13, 10 and 243 copies of AKI, beta-actin and DWV respectively.

### Statistical analyses

Statistical analysis and data handling was carried out in Microsoft Excel and R [50]. All figures in this paper were generated in R. All statistical tests were non-parametric as the data was non-normal and heteroscedastic due to zero-clumping. All tests were carried out on log_10_ viral copies. Significance testing between two independent groups was done using Mann-Whitney-U Test (Wilcoxon Rank Sum Test) (W) and tests for more than two groups were done using Kruskal-Wallis test (KW). Viral prevalence in the colonies were calculated online using EpiTools pooled prevalence calculator (http://epitools.ausvet.com.au) [51] using method 2 [52], [53]. For a few colonies, all 10 monthly sub-samples were positive or negative rendering them unsuitable for prevalence calculation. For five of these colonies, viral titres in six extra bees were analysed individually.

## Results

### Survival and varroa levels

Of the 23 hives, all except seven hives survived to spring 2012. One untreated hive (C02) was lost in September 2011 due to American foulbrood and therefore not an overwintering loss. Three hives from the organic group (A04, A05, A09), one hive from the pyrethroid group (B09) and two hives from the untreated group (C01, C03) died over winter. The mean bee count across 176 samples from eight months (excluding dead hives) was 231±68 bees ranging from 112 to 484 bees. The mean varroa mite count across 176 samples from eight months (excluding dead hives) was 8±23 mites ranging from 0 to 242 mites.

**Figure 1:**
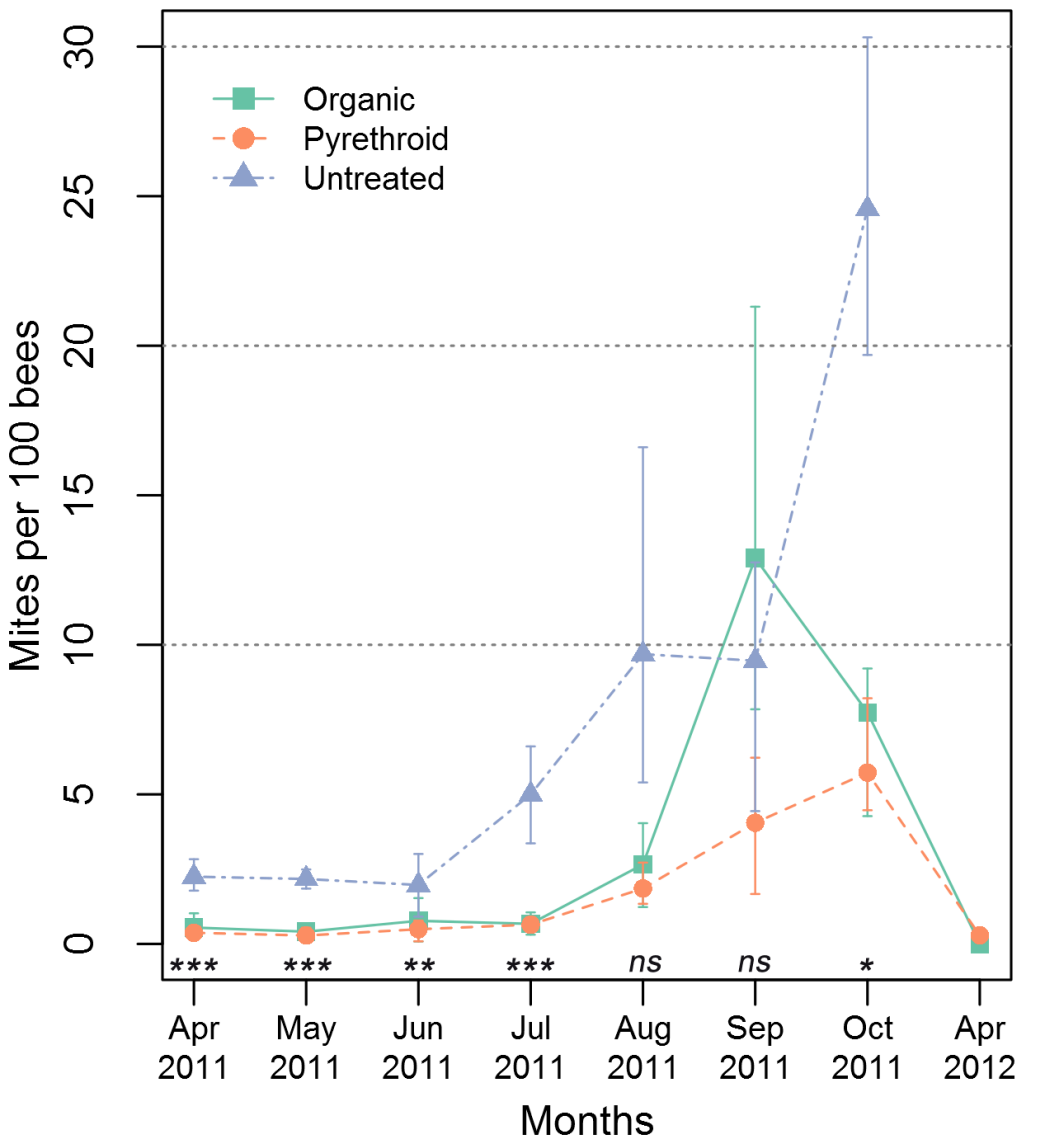
Varroa mite indices for the three treatment groups over eight months. Mite indices were computed using sum of bees and sum of mites in each treatment group. These are not mean of mite indices of samples. Error bars show standard error. Error bars were computed using n as follows: Organic group (n = 11 colonies except Apr 2012 where n = 8), Pyrethoid group (n = 9 except Apr 2012 where n = 8), Untreated group (n = 3 except Oct 2011 where n = 2 and Apr 2012 where n = 0). Asterix indicate significance (Wilcoxon test) between treated and untreated colonies (* P<0.05, ** P<0.01, *** P<0.001).

The mite indices for three treatment groups are shown in Figure 1. The mite index was significantly different between treatment groups for the months of April 2011 (KW P = 0.023), May 2011 (KW P = 0.0098) and July 2011 (KW P = 0.02). The curves for treated organic and pyrethoid groups showed similar trend except in September where the organic group showed a sharp rise. This drastic rise in mites for September was mainly based on five colonies from one organic apiary. Mite indices in treated colonies were significantly lower than untreated colonies for all months except Aug and Sep (Figure 1). The mite load in the untreated group continued to rise until they collapsed. The untreated group was not treated the year before, hence, they started the season with more mites than the two treated groups.

### Viral titres

**Figure 2:**
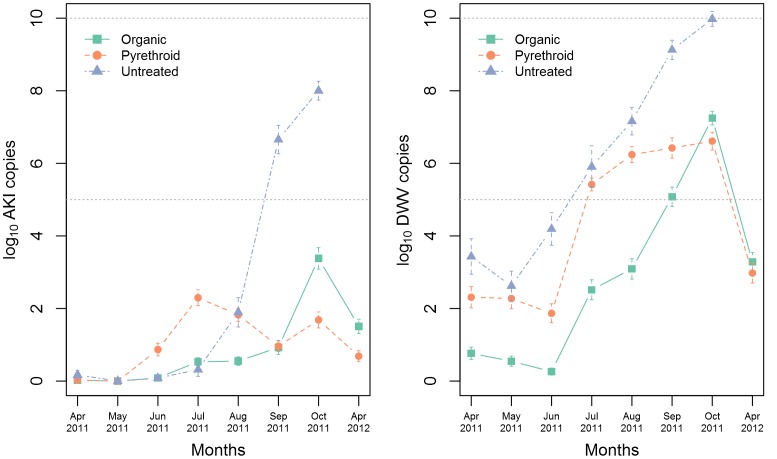
Mean viral titres in bees (subsamples) for three treatment groups across eight months for AKI (left) and DWV (right). Number of sub-samples in the organic group (n = 110 except in Apr 2012 where n = 80), pyrethroid group (n = 90 except in Apr 2012 where n = 80), untreated group (n = 30 except in Oct 2011 where n = 20). Error bars show standard error. Untreated colonies showed significantly higher AKI titres in May and July and significantly higher DWV titres in June and Sep compared to treated colonies.

DWV titre was generally higher than AKI titre in all three treatment groups (Figure 2). For AKI, the viral titre rose from April to July in all three treatment groups. In organic and pyrethroid groups, AKI titres decreased in connection with the varroa treatment during August, but then increased again. In the untreated group, AKI titre peaked in September. For DWV, all groups displayed a steady rise in viral titre throughout the season. Viral titres in mites also showed a rising trend over the season (Figure S2). AKI titres in treated colonies were significantly different (Wilcoxon) from untreated colonies only in May and July. DWV titres in treated colonies were significantly different (Wilcoxon) from untreated colonies only June and Sep.

**Figure 3:**
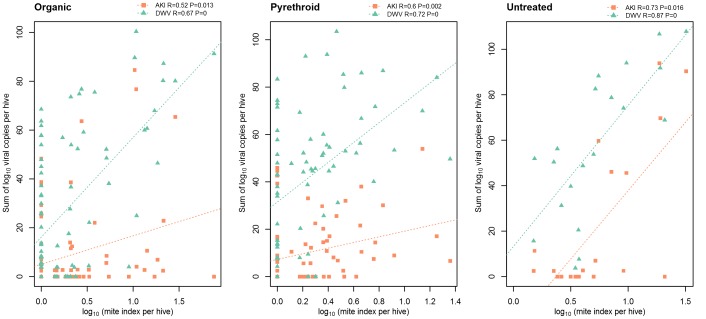
Correlation of mite index versus viral copies for the three treatment methods. Organic (n = 85), Pyrethroid (n = 71), Untreated (n = 20). For all three treatment groups and for both viruses, the correlations are highly significant.

The sum of viral titres in the sub-samples of AKI and DWV copies per hive was correlated to mite index (mites per 100 bees) per hive (Figure 3). In all three treatment groups, viral copies were significantly (P<0.01) correlated to mite index.

### Prevalence of diseased bees

Splitting each sample into 10 subsamples allowed us to determine the prevalence of virus-infected bees using pooled prevalence calculations. Depending on the time of the year, we find huge variation in the number of infected bees. For AKI, we had zero of 230 (0%) sub-samples infected in May to 130 of 220 (59%) sub-samples infected in October. For DWV, we had 65 of 230 (28%) sub-samples infected in June up to 216 of 220 (98%) infected sub-samples in October. Of the dying colonies, all (100%) sub-samples were infected with DWV while 56 of 60 (93%) sub-samples were infected with AKI in October.

**Figure 4:**
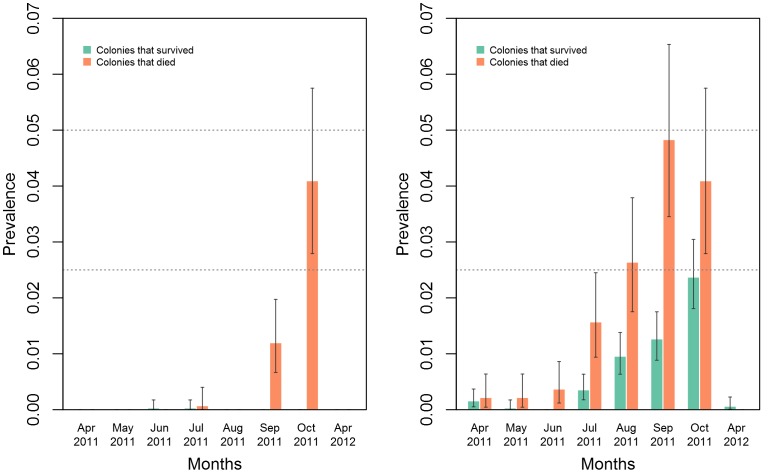
Prevalence of workers infected with greater than 10^7^ copies of AKI and DWV viruses across eight months in subsamples (n = 160) from colonies that survived (n = 16) and subsamples (n = 70 except Oct 2011 where n = 60 and Apr 2012 where n = 0) from colonies that died (n = 7 except Oct 2011 where n = 6 and Apr 2012 where n = 0) during the experiment. Error bars show 2.5% and 97.5% confidence intervals.

In order to determine the fraction of workers with detrimental infection, our solution was to include only sub-samples with titres above a defined threshold. We considered sub-samples of 20 bees with viral titres above 10^7^ copies an indication of at least one individual bee within the sub-sample being diseased. We compared the prevalence of diseased bees for every month in colonies that died to the prevalence of diseased bees in colonies that survived the following winter. Monthly prevalence was calculated based on the number of sub-samples showing greater than 10^7^ copies by combining all sub-samples from surviving (n = 160) and dying (n = 70 except October n = 60) colonies, respectively. Colonies that died showed significantly higher prevalence of AKI and DWV infected bees during the months of September and October (Figure 4).

The pooled prevalence calculator is unable to determine the prevalence when all 10 sub-samples of a colony are positive. But in order to directly verify the prevalence of bees with viral titres above 10^7^ copies, we tested individual bees from four colonies in September (A02, A03, A04, A09) and four colonies in October (A02, A03, A04, A05) (Figure S8). This included two colonies that survived (A02, A03) and three colonies that died (A04, A05, A09). For AKI, we found considerable variation amongst the tested individuals. All individual bees from the two colonies that survived (A02, A03) had viral titres below 3500 copies. In contrast, the individuals from the three colonies that died had AKI titres ranging from zero to 6.9×10^9^ copies. For DWV, the picture is quite different. In the two colonies that survived, none of the 12 bees had viral titres above 10^7^ copies in September. However, by October, one of the surviving colonies had two of six bees with titres above 10^7^ copies. In the dying colonies, 12 of 24 bees were above 10^7^ copies. The viral copies observed in individual bees cannot be directly compared to pooled sub-samples due to differences in methodology.

### Case study

**Figure 5:**
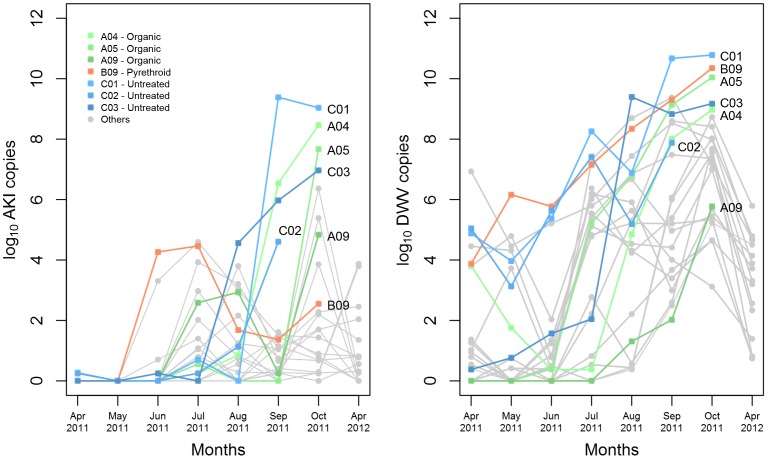
AKI and DWV titres (sum of subsample titres) of colonies that collapsed (coloured, ▪) in comparison to surviving colonies (gray, •) for each month. Green colour denotes organic group, red colour denotes pyrethroid group, blue colour denotes untreated group and gray colour denotes surviving colonies regardless of treatment. Hive C02 was destroyed in September due to American foulbrood. Colonies that died had significantly higher AKI titres in Sep and Oct compared to colonies that survived. Colonies that collapsed had significantly higher DWV titres for all months except July compared to colonies that collapsed.

We investigated the mite infestation and viral titres of seven colonies that died compared to 16 colonies that survived winter. The overall mite index of the colonies that died was significantly higher (W P = 5.8×10^−5^) than those colonies remaining alive. The mite index is shown in Table 1. The September and October AKI titres of the colonies that collapsed were significantly higher than those that survived (W P = 7.7×10^−8^). DWV titres for colonies that collapsed compared to those that survived were significantly higher (W P<0.05) during all months except July. From Figure 5, it is clear that during the months of September and October, the AKI titres of the dying colonies were substantially higher with the exception of colony A09, which however showed a sudden rise in AKI titre in October. For DWV, a similar scenario exists. However, colony A09 has one of the lowest titres. So it seems unlikely that DWV was the cause of mortality in this colony, but the sudden rise of AKI in October could indicate that the colony had contact with an infected source. AKI titre in colony A05 remained low or zero throughout the season until September and then showed a drastic rise to 10^8^ copies in October. This also coincided with a huge rise in mite index in September possibly due to influx of mites from the surroundings. Colony C02 showed rapidly rising titres until September, when we were forced to destroy it due to an outbreak of American foulbrood.

**Table 1:** Varroa mite index (mites per 100 bees) from surviving and dead colonies.

Month	Surviving (Treated)	Dead (Treated)	Dead (Untreated)	Signif¤
APR	0.2	1.6	2.3	**
MAY	0.3	0.7	2.2	*
JUN	0.2	2.3	2.0	*
JUL	0.6	1.2	5.0	ns
AUG	1.5	5.3	11.0	ns
SEP	4.4	29.2	8.6	ns
OCT	6.0	8.9	25.0#	ns
n	16	4	3#	

All surviving colonies (n = 16) were treated. Four colonies that died over the winter were treated. All untreated colonies died over winter.

# n = 2 in October. Despite the treatment, mite infestation level increased, especially in the succumbing colonies. This could be due to ineffective treatment or subsequent mite reinvasion.

¤ Significant difference in mite index between surviving and dead colonies (* P<0.05, ** P<0.01, *** P<0.001).

Comparing the viral titres in the varroa mites to titres in bees, we find considerable discrepancy. There are several examples of cases where the mite sample was positive while the accompanying bees were negative. Similarly, we have also found cases where mite sample was negative while the bee subsamples were positive. Viral titres in the mite samples across months are shown in Figure S2.

## Discussion

The drastic loss of honey bee colonies in recent years has seriously impacted beekeeping worldwide. Although causes remain indecisive, varroa mites and associated viruses are often mentioned as likely culprits. Therefore, we decided to investigate the relationship between colony survival, varroa treatment and viral load. Our results suggest that the methods used do not always yield satisfactory results. Mite numbers and viral titres in September and October continued to rise in several apiaries despite the treatment in August. This had major consequences for colony health as seven colonies out of 23 were lost over the winter period. Of the seven colonies that died during the experiment, one was destroyed due to AFB, but the death of all other colonies coincided with the sudden increase in viral titres in autumn. We consider the remaining six colonies to have died as a result of varroa-virus interaction.

For beekeepers and scientists alike, estimating varroa load in colonies is difficult and cumbersome [46]. The mite index defined as the number of mites per 100 bees is a commonly used measurement of mite load in the colony. The rise in mite load from spring to summer is slow due to increased brood production. The steep rise in varroa mites from July till September and the decreasing bee population in the same period results in considerable variation in mite index over time. Using mite index to determine the mite load demands several samplings over time and remains impractical for commercial beekeeping. Therefore, many beekeepers treat all hives on a set schedule irrespective of mite index. A large German bee monitoring project [2] reported mite index for October in surviving colonies (3.2–3.6, n = 2906) to be significantly different from collapsing colonies (14.6–16.5, n = 368). In our study, the mite index in September for the six colonies that we presume died of varroa/viruses were 0, 3, 5, 18, 28, and 74 which progressed to 0, 15, 18, 31, 9 and 10 in October respectively (Figure S7). The reduction in mite index for the last two colonies from September to October is possibly due to treatment. We see a more uniform distribution of the mites in October as compared to September, indicating flow of mites between colonies in the vicinity of each apiary.

According to the established varroa-virus model [40], [44], increasing varroa population in the colony leads to higher transmission of viruses amongst the bees. Build-up of DWV leads to high proportion of bees with reduced survivorship impacting colony survivorship. Colonies may recover from a large DWV mite population if mites are removed early enough, allowing successful overwintering of bees. As a consequence of the beekeepers' annual treatment, the bees, the mites and DWV co-exist from one season to the next. In stark contrast, ABPV rapidly kills infected pupae preventing the developing bee to emerge, therefore ABPV vectoring mites are unlikely to reproduce successfully. Collapse of a colony due to ABPV occurs only if a large mite population is present when the virus is introduced, according to the varroa-virus model [44].

The German bee monitoring project [2] further considered the impact of viral infections on colony survivorship. In particular, they noticed that the presence of DWV was a good indicator for the mortality of colonies. A weaker link was noticed for ABPV and no significant relationship was noted for KBV. An American study [7] reported IAPV to be a strong predictor for CCD colonies. A study in Hawaii also showed strong increase in titre and prevalence of DWV in colonies as a result of varroa introduction, but no such relationship was noted for ABPV, KBV or IAPV viruses (Martin, 2012). In our study, we find a link between varroa infestation as well as viral titres for both AKI and DWV, to colony mortality. In September and October, most dying colonies showed higher AKI and DWV titres than surviving colonies (Figure 5). The sum of viral titres from all subsamples is plotted in Figure 5, and it can be noted that there is considerable variation between the subsamples (Figure S6). Hence, examining samples of less than 200 bees could lead to an uncertain estimate of the titre. For the survival of the bee colony, the number of diseased individual bees is a better indicator than the overall viral titre. With the help of the 10 subsamples of 20 individual bees, it is possible to infer the prevalence of diseased bees. The observed prevalence of AKI infection in colonies during early spring was extremely low, indicated by only four positive sub-samples out of 460 in April and May 2011. However, later in the season, the prevalence of AKI-positive subsamples increased steadily (Figure S3). It is more informative only to consider sub-samples that exceed our threshold value of 10^7^ copies, representing diseased bees (Figure S4). Since all 10 subsamples were positive for several of the colonies, it was not possible to calculate the prevalence for each colony. Therefore, we considered the subsamples of all surviving colonies and the dying colonies as two separate pools, from which we could calculate the month-wise prevalence of diseased individuals (Figure 4). The prevalence of AKI-diseased bees peaked in October reaching 4% of the workers in the dying colonies compared to zero percent in surviving colonies. For DWV, the difference in October is less pronounced at 4% in dying colonies compared to 2.5% in survivors. This makes DWV less predictive of colony death than AKI. The 48 bees that were analysed individual from surviving and dying colonies (Figure S8) confirms that considerable variation exists among individuals. The viral copies between individual bees and pooled subsamples are not directly comparable due to difference in methodology. The samples from non-surviving colonies indicate that only 4% of the workers exhibit detrimental viral titres. This suggests a threshold level of viral prevalence beyond which a domino effect sets in, probably mediated by varroa mites shifting host [44], [54] leading to colony death.

Our results support the notion that high varroa load leads to build-up of high viral titres. As an exception, no varroa mites were detected in the bee samples for the entire year in colony A09. Yet, in October 2011, this colony suddenly exhibited increased level of virus prevalence for both AKI and DWV before collapsing in winter. This suggests that viral infections can spread between colonies even at low mite loads. The observed absence of varroa mites may be due to inadequate sampling. This is a typical problem when one has to balance between optimal sampling size and influencing survival of the studied colonies due to bee and mite removal. Too large samples or too frequent sampling will remove bees and varroa mites from the studied colonies. Beyond the influence of sampling on the final result, there is also the aspect of available time and money.

Based on the experiments done here, it is clear that a uniform sampling size across the season is not ideal. Much more can be learned from analysing biological replicates. We used a standard subsample size of 20 bees, based on the assumption that few bees have high viral load in spring and expected a build up over the season. In May, none of the subsamples were positive. In September and October, we observed all 10 subsamples to be positive for several colonies. In other words, subsamples of 20 bees were too large. Ideally, about half the pools are to be positive and half negative to achieve a reliable estimation of prevalence. Since the number of diseased bees was unknown, pools of 20 individuals were used throughout. In retrospect, it might have been wiser to split the analysis into several phases. An initial screening of two subsamples of 20 bees each followed by increased or decreased pool size depending on the initial results.

This study shows a clear trend of rising viral titres in bee populations over the course of a season from spring to autumn. Despite the application of acaricide, varroa mites and viral titres continued to rise while remaining below the levels observed in the untreated group. In many cases, the treatment only helped to control varroa loads and viral titres to a limited extend, and led to no significant reduction in the prevalence of virus-sick bees. In part, this may be explained by the recent findings [47], [48], that show negative impact of acaricides on honey bee immunity. Adding to this effect of acaricides, the observed failure of the treatment to reduce varroa numbers in several colonies might have led to immuno-suppression syndrome as observed in other studies [18], [55]. Our findings differ from a previous study [56], where an immediate drop of DWV titre was observed following varroa treatment. It has been noted in several studies that DWV reduces the lifespan of overwintering workers often in connection with varroa mites [1], [3]. DWV has also been suggested to lead to colony losses independent of varroa infestation possibly in synergy with an uncharacterised stress factor [4]. Thus, we hypothesise that the effect of treatment may constitute this stress factor.

AKI and DWV viruses play a major role in declining colonies in Denmark. Varroa mites are the driving factor that has led to an upsurge in viral titres. Beekeepers implement various treatment regimes against varroa mites to prevent colony losses, which in turn lead to persistence viral infection in the colonies. The immune-suppression resulting from pesticide usage in the hive linked with the higher prevalence of viral infections may result in sudden colony loss. Beekeepers often experience low efficacy after years of recurring treatments. This subsequently leads to more aggressive treatment methods resulting in an unsustainable warfare on varroa mites, also detrimental to bee health. One practical solution to this malady would be selective treatment of colonies exhibiting high mite load. Knowledge of mite and virus levels in bee colonies is essential to establish a threshold beyond which colony loss is unavoidable. Ideally this threshold can be use in selection programs to identify and multiply colonies for which yearly treatment is not required. In Germany, the ‘Arbeitsgemeinschaft Toleranzzucht’, a co-operation of queen breeders in collaboration with researchers are working towards achieving this goal (www.toleranzzucht.de) [57].

## Supporting Information

Figure S1 **Dynamic range of quantification for AKI, beta-actin and DWV primers.** Correlation (R^2^) and reaction efficiency (E) for each primer pair are shown. Error bars show standard deviation based on two replicates each on eight plates (n = 16 for each point except * where n = 10).
(TIFF)

Figure S2 **Viral titres in varroa mites in three treatment groups across eight months (Left: AKI, Right: DWV).** Error bars show standard error.
(TIF)

Figure S3 **Proportion of bee sub-samples showing presence of viral infection in three treatment groups across eight months.** Left: Proportion of bee sub-samples showing AKI infection. The organic and pyrethroid groups are reduced by treatment while untreated group continues to rise over the season. Right: Proportion of bee sub-samples showing DWV infection. All groups start at a higher infection proportion. All groups show rising proportion of infection. Treatment does not seem to suppress the spread of infection.
(TIF)

Figure S4 **Proportion of bee sub-samples showing greater than 10^7^ viral copies in three treatment groups across eight months.** Left: AKI and Right: DWV.
(TIF)

Figure S5 **Geographical locations of colonies used in this study.** Inset bottom: Bornholm is an island located about 140 km east of Denmark.
(TIF)

Figure S6 **AKI and DWV titre data is shown in log_10_ copies after C_t_ 34 cut-off.** Sampling months are shown row-wise which includes 10 subsamples, varroa-free subsample as 0 (if sampled) and varroa as VAR (if present). Colonies are shown in columns. Contiguous background fill colour for the colony names represent apiaries. The colour of the colony text denotes treatment category. Green - Organic, Red - Flumethrin, Blue - Untreated. Viral titres are colour-coded into four groups. Zero or null virus is not coloured. 10–10^4^ copies or low-level infection is coloured green, 10^4^–10^7^ copies or medium-level infection is coloured yellow and greater than 10^7^ copies is coloured red showing serious damaging infection.
(TIF)

Figure S7 **Raw mite counts, bee counts and mite indices are shown month-wise for all colonies.** Colony names and colours are as explained for figure S6. The colour scheme is applied independently in the three tables. Colonies that died are marked as ‘x’.
(TIF)

Figure S8 **Log_10_ AKI and DWV titres for 48 bees that were individually analysed.** ‘S’ denotes colonies that survived while ‘D’ denotes colonies that died.
(TIF)

Table S1 **List of primers used in this study.** Outer primers were used to prepare standard curves.
(DOC)
