# Supplementary figures and images for: Varroa-Virus Interaction in Collapsing Honey Bee Colonies
Source: PLoS One. 2013 Mar 19;8(3):e57540. doi: 10.1371/journal.pone.0057540 (PMC3602523; doi:10.1371/journal.pone.0057540)

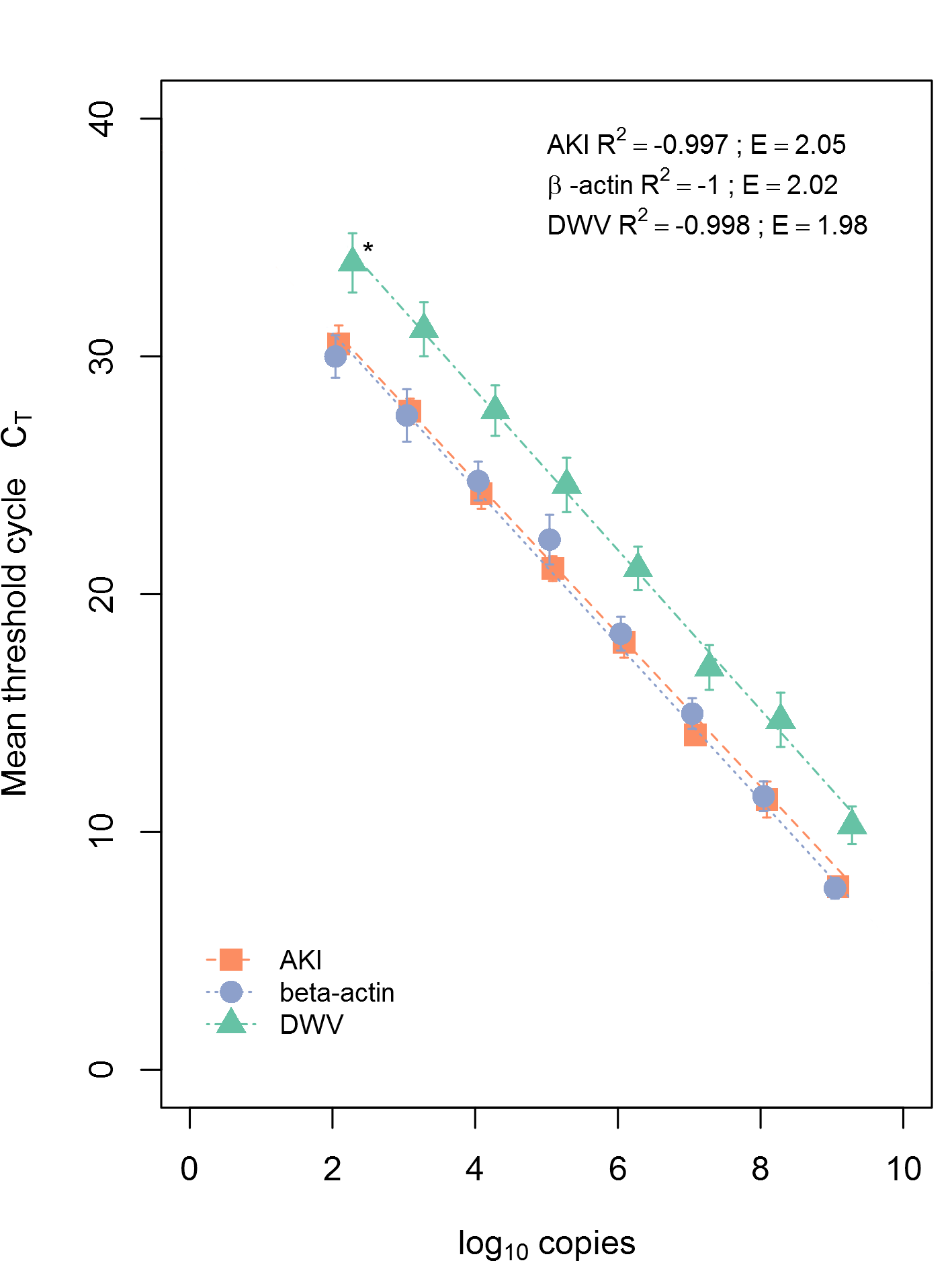

Supplement: Figure S1 — Dynamic range of quantification for AKI, beta-actin and DWV primers. Correlation (R2) and reaction efficiency (E) for each primer pair are shown. Error bars show standard deviation based on two replicates each on eight plates (n = 16 for each point except * where n = 10). (TIFF) [file pone.0057540.s001.tif]

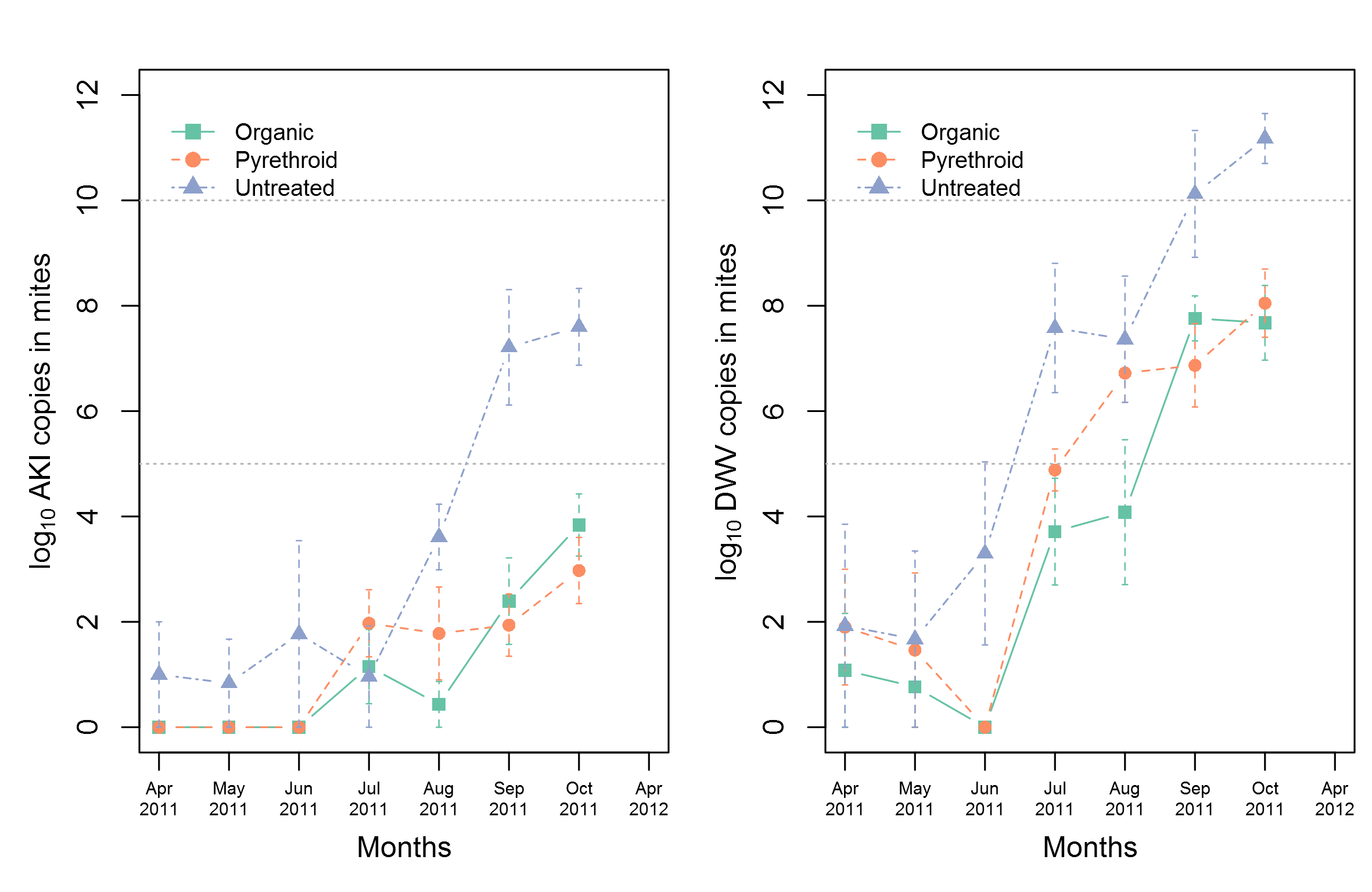

Supplement: Figure S2 — Viral titres in varroa mites in three treatment groups across eight months (Left: AKI, Right: DWV). Error bars show standard error. (TIF) [file pone.0057540.s002.tif]

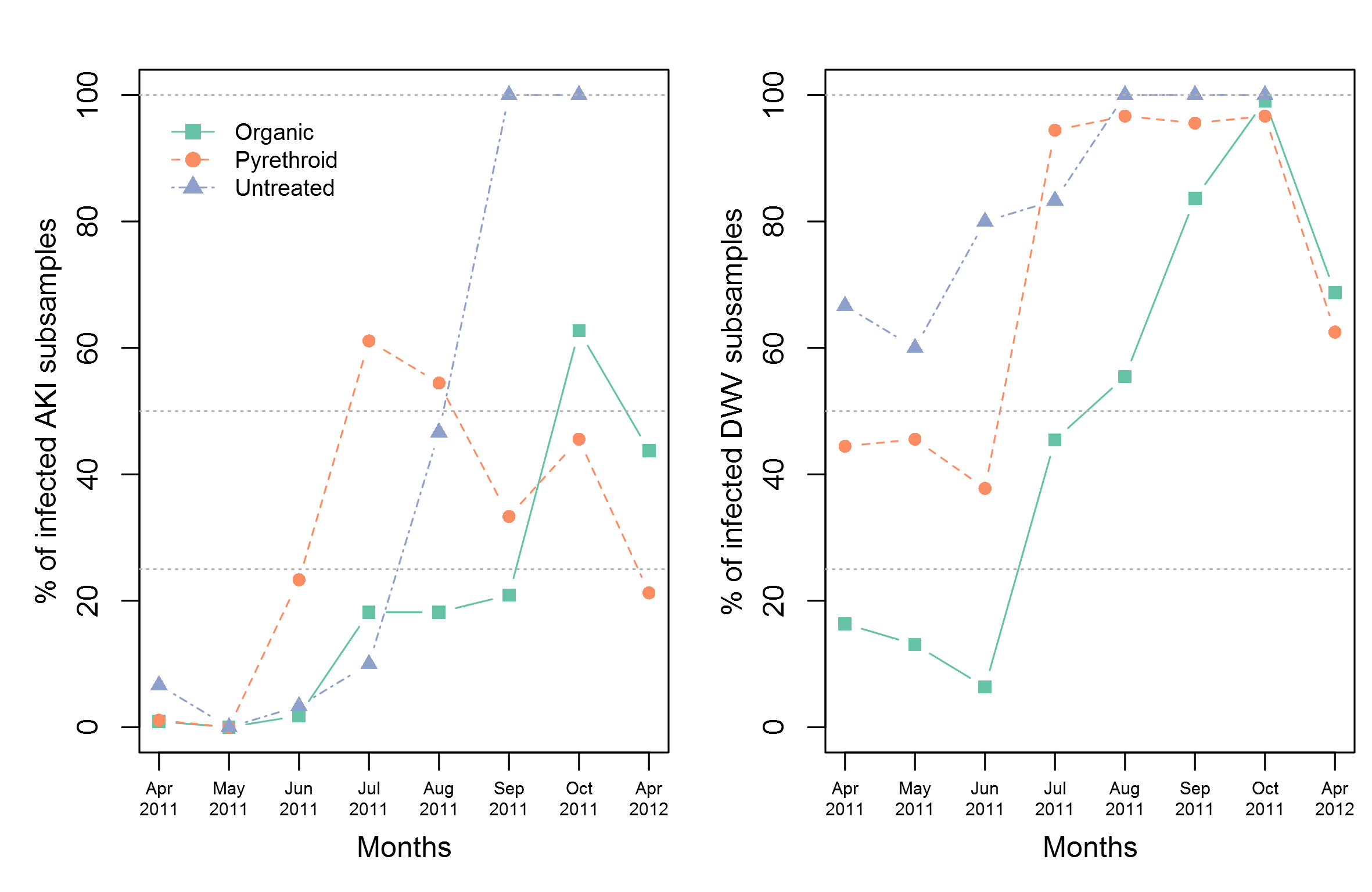

Supplement: Figure S3 — Proportion of bee sub-samples showing presence of viral infection in three treatment groups across eight months. Left: Proportion of bee sub-samples showing AKI infection. The organic and pyrethroid groups are reduced by treatment while untreated group continues to rise over the season. Right: Proportion of bee sub-samples showing DWV infection. All groups start at a higher infection proportion. All groups show rising proportion of infection. Treatment does not seem to suppress the spread of infection. (TIF) [file pone.0057540.s003.tif]

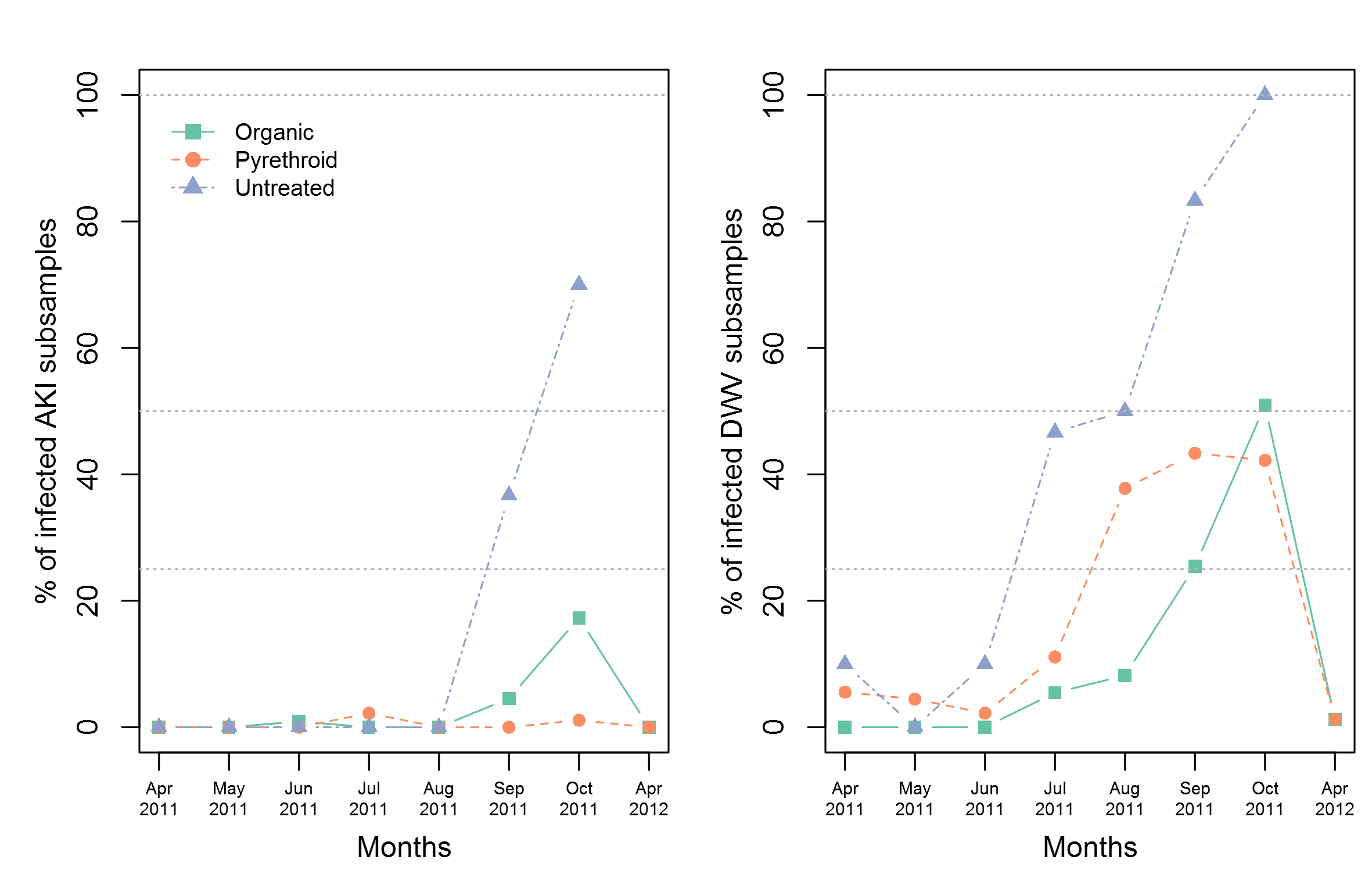

Supplement: Figure S4 — Proportion of bee sub-samples showing greater than 107 viral copies in three treatment groups across eight months. Left: AKI and Right: DWV. (TIF) [file pone.0057540.s004.tif]

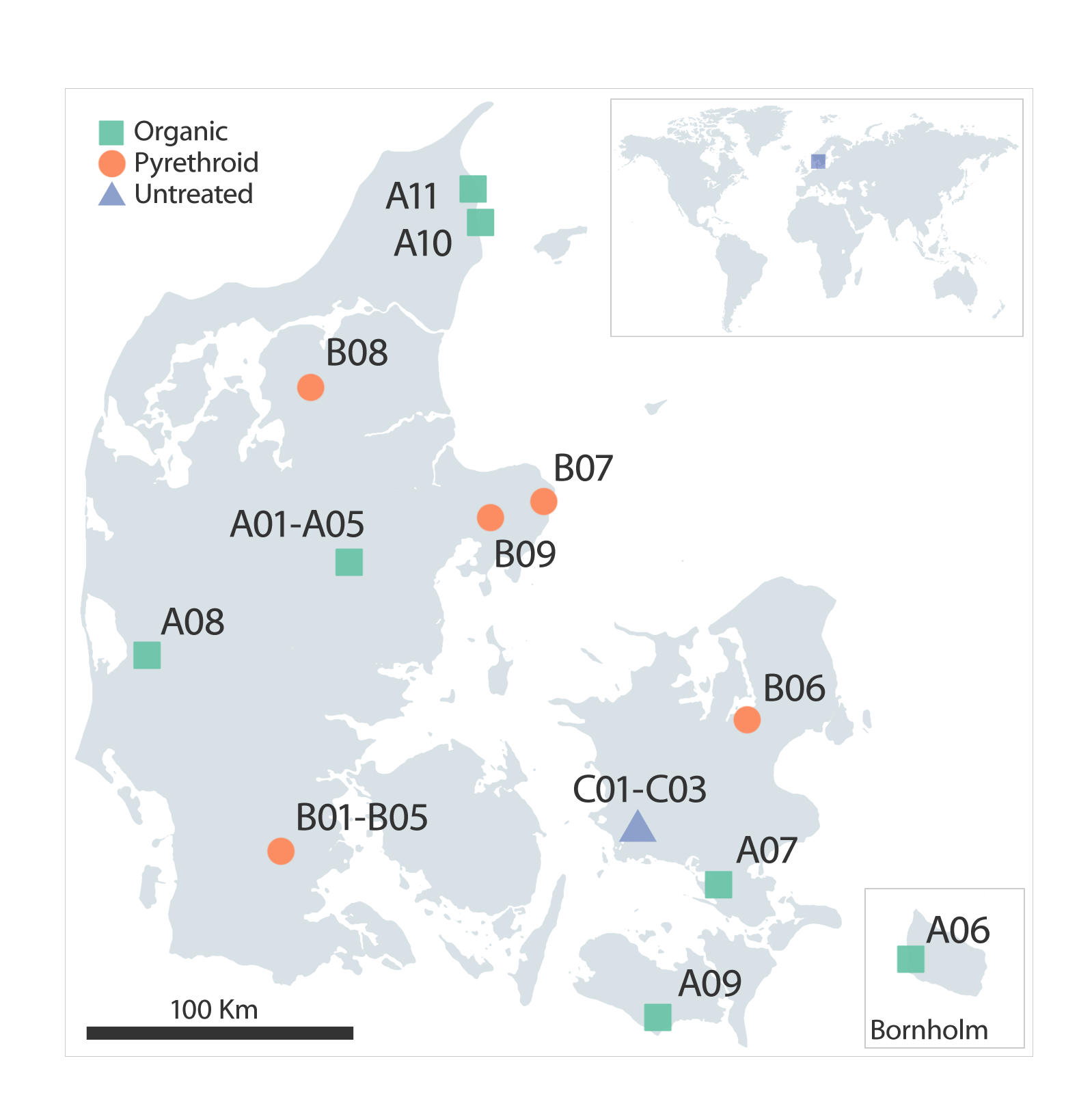

Supplement: Figure S5 — Geographical locations of colonies used in this study. Inset bottom: Bornholm is an island located about 140 km east of Denmark. (TIF) [file pone.0057540.s005.tif]

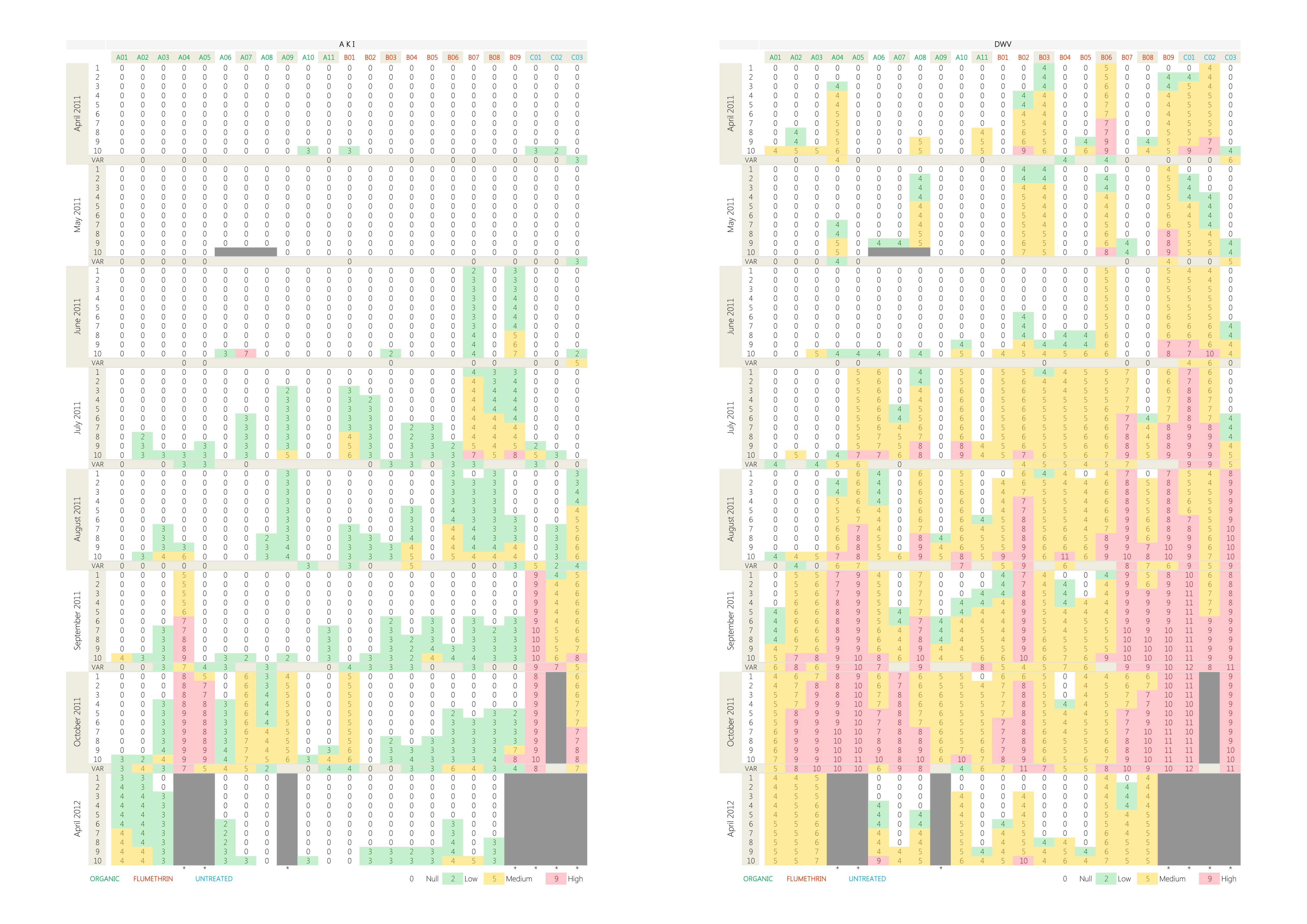

Supplement: Figure S6 — AKI and DWV titre data is shown in log10 copies after Ct 34 cut-off. Sampling months are shown row-wise which includes 10 subsamples, varroa-free subsample as 0 (if sampled) and varroa as VAR (if present). Colonies are shown in columns. Contiguous background fill colour for the colony names represent apiaries. The colour of the colony text denotes treatment category. Green - Organic, Red - Flumethrin, Blue - Untreated. Viral titres are colour-coded into four groups. Zero or null virus is not coloured. 10–104 copies or low-level infection is coloured green, 104–107 copies or medium-level infection is coloured yellow and greater than 107 copies is coloured red showing serious damaging infection. (TIF) [file pone.0057540.s006.tif]

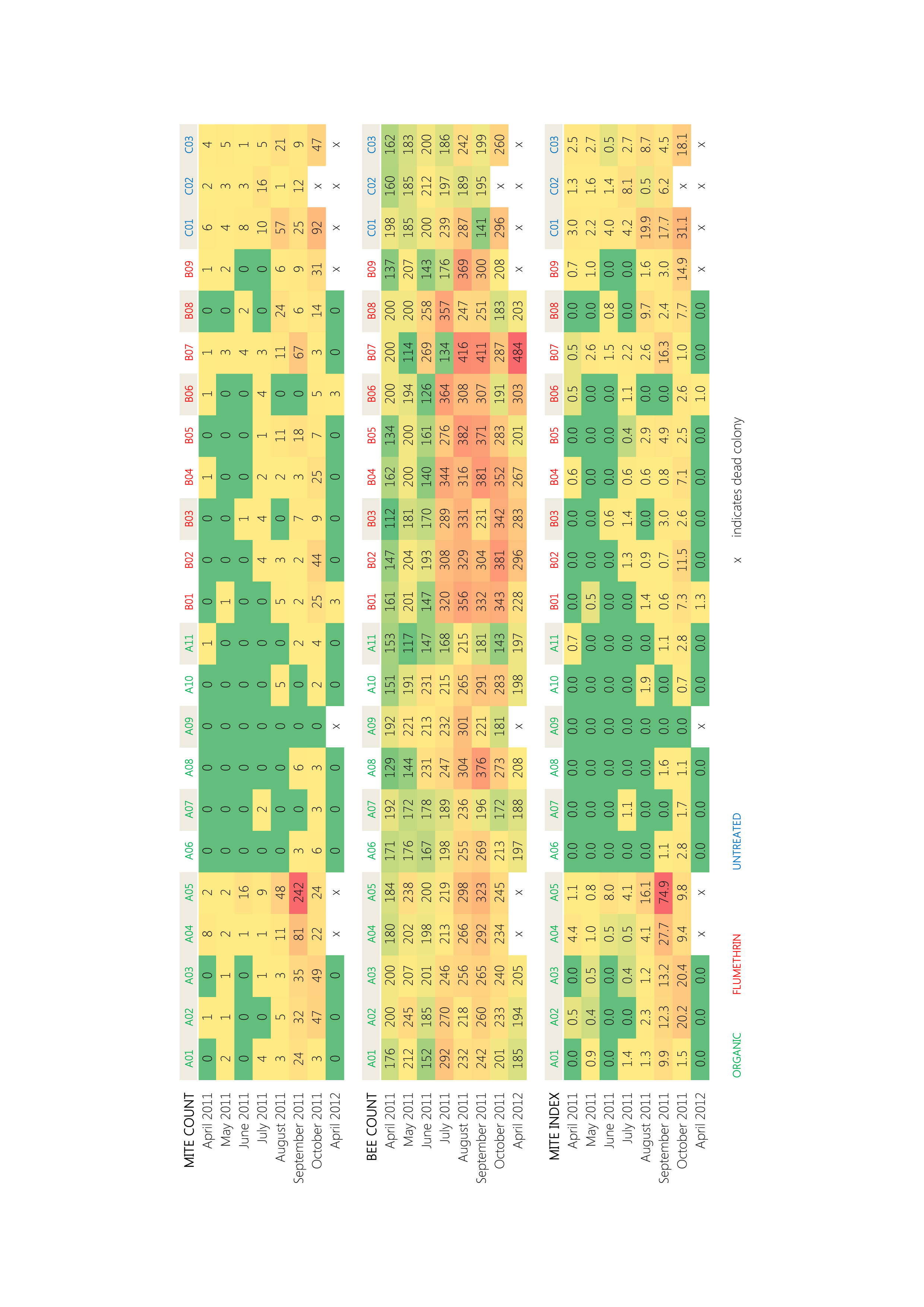

Supplement: Figure S7 — Raw mite counts, bee counts and mite indices are shown month-wise for all colonies. Colony names and colours are as explained for figure S6. The colour scheme is applied independently in the three tables. Colonies that died are marked as ‘x’. (TIF) [file pone.0057540.s007.tif]

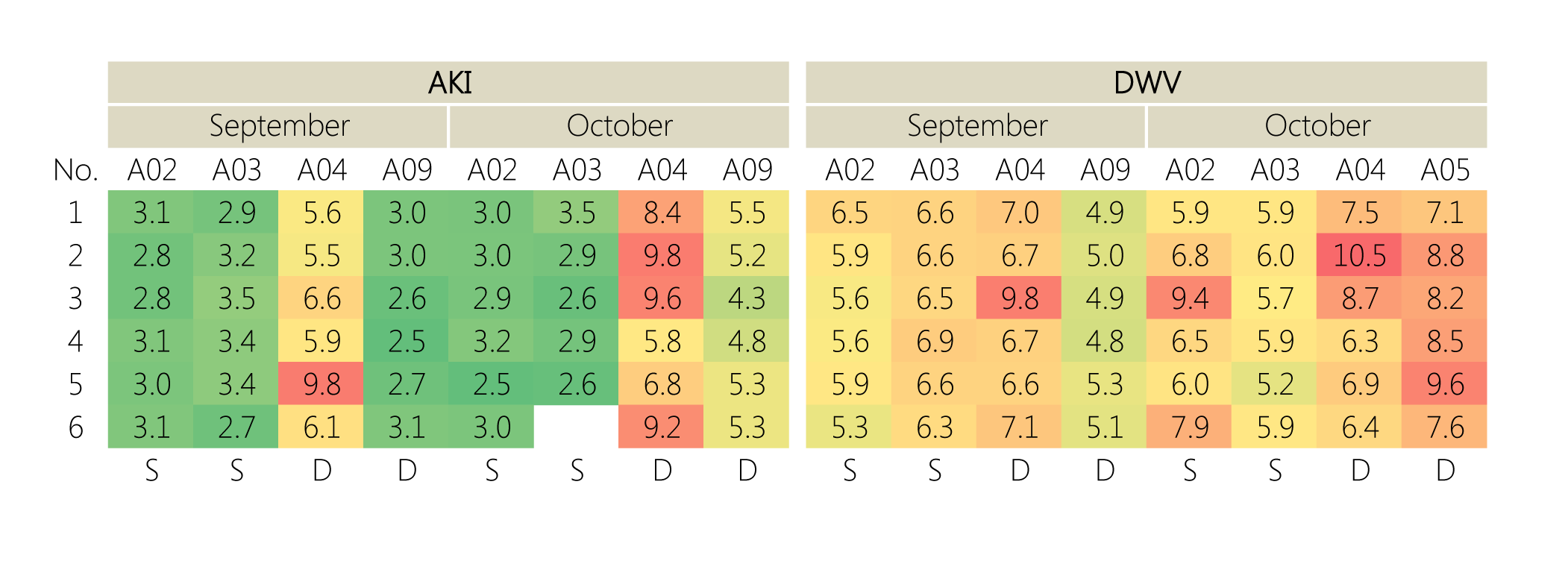

Supplement: Figure S8 — Log10 AKI and DWV titres for 48 bees that were individually analysed. ‘S’ denotes colonies that survived while ‘D’ denotes colonies that died. (TIF) [file pone.0057540.s008.tif]
