# Supplementary material for: Varroa-Virus Interaction in Collapsing Honey Bee Colonies
Source: PLoS One. 2013 Mar 19;8(3):e57540. doi: 10.1371/journal.pone.0057540 (PMC3602523; doi:10.1371/journal.pone.0057540)
Supplement: Table S1 — List of primers used in this study. Outer primers were used to prepare standard curves. (DOC) [file pone.0057540.s009.doc]

| Primer | Sequence | Amplicon Length | Reference |
| --- | --- | --- | --- |
| DWV1* | TTTGCAAGATGCTGTATGTGG | 395 | [1] |
|  | GTCGTGCAGCTCGATAGGAT |  |  |
| DWV3 | GGATGTTATCTCCTGCGTGGAA | 69 | [2] |
|  | CTTCATTAACTGTGTCGTTGATAATTG |  |  |
| AKI2* | GGATGCCCTATTTAGGGTGAG | 362 | (Francis et al., 2012, Unpubl.) |
|  | CCTCAATGTGGTCAATGAGTACG |  |  |
| AKI | CTTTCATGATGTGGAAACTCC | 100 | [3] |
|  | AAACTGAATAATACTGTGCGTA |  |  |
| beta-actin1* | AGGAATGGAAGCTTGCGGTA | 181 | [4] |
|  | AATTTTCATGGTGGATGGTGC |  |  |
| beta-actin4 | TGCCAACACTGTCCTTTCTGGAGGT | 96 | (Francis et al., 2012, Unpubl.) |
|  | TTCATGGTGGATGGTGCTAGGGCAG |  |  |
| Vbeta-actin1* | CGACGGTCAGGTCATCAC | 243 | [5] |
|  | GTTGAGGGAGCCAAAGAGG |  |  |
| Vbeta-actin2 | GTTCATCGGAATGGAGTCATGCGGT | 108 | (Francis et al., 2012, Unpubl.) |
|  | CCAGAGAGAACGGTGTTAGCGTACAGA |  |  |

*Outer primers

Cycling conditions for AKI outer primers (AKI2) were 3 min at 94°C, 35 cycles of 15s at 94°C; 35s at 60°C; 35s at 72°C followed by 10 min at 72°C. The cycling conditions for DWV outer primers (DWV1) were 3 min at 94°C, 35 cycles of 15s at 94°C; 35s at 56°C; 35s at 72°C followed by 10 min at 72°C. The temperature profile for all three inner primers (AKI, beta-actin4, DWV3) were as follows: 2 min at 50 °C, 10 min at 95 °C and 40 cycles of 15s at 95 °C; 1 min at 60 °C. Temperature profile for the dissociation curve analysis was: 15s at 95 °C, 15s at 60 °C and 15s at 95 °C.

**References**

1. Tentcheva D, Gauthier L, Jouve S, Canabady-Rochelle L, Dainat B, et al. (2004) Polymerase Chain Reaction detection of deformed wing virus (DWV) in Apis mellifera and Varroa destructor. Apidologie 35: 431-439.

2. Gauthier L, Tentcheva D, Tournaire M, Dainat B, Cousserans F, et al. (2007) Viral load estimation in asymptomatic honey bee colonies using the quantitative RT-PCR technique. Apidologie 38: 426-U427.

3. Francis RM, Kryger P (2012) Single Assay Detection of Acute Bee Paralysis Virus, Kashmir Bee Virus and Israeli Acute Paralysis Virus. Journal of Apicultural Science 56: 137-146.

4. Chen YP, Zhao Y, Hammond J, Hsu HT, Evans J, et al. (2004) Multiple virus infections in the honey bee and genome divergence of honey bee viruses. Journal of Invertebrate Pathology 87: 84-93.

5. Locke B, Forsgren E, Fries I, de Miranda JR (2012) Acaricide Treatment Affects Viral Dynamics in Varroa destructor-Infested Honey Bee Colonies via both Host Physiology and Mite Control. Applied and Environmental Microbiology 78: 227-235.
